# Role of the Intracellular Sodium Homeostasis in Chemotaxis of Activated Murine Neutrophils

**DOI:** 10.3389/fimmu.2020.02124

**Published:** 2020-09-08

**Authors:** Karolina Najder, Micol Rugi, Mégane Lebel, Julia Schröder, Leonie Oster, Sandra Schimmelpfennig, Sarah Sargin, Zoltán Pethő, Etmar Bulk, Albrecht Schwab

**Affiliations:** ^1^Institute of Physiology II, University Hospital Münster, Münster, Germany; ^2^University of Florence, Florence, Italy; ^3^University of Sherbrooke, Sherbrooke, QC, Canada

**Keywords:** neutrophil, chemotaxis, intracellular sodium, NCX1, TRP channels

## Abstract

The importance of the intracellular Ca^2+^ concentration ([Ca^2+^]_i_) in neutrophil function has been intensely studied. However, the role of the intracellular Na^+^ concentration ([Na^+^]_i_) which is closely linked to the intracellular Ca^2+^ regulation has been largely overlooked. The [Na^+^]_i_ is regulated by Na^+^ transport proteins such as the Na^+^/Ca^2+^-exchanger (NCX1), Na^+^/K^+^-ATPase, and Na^+^-permeable, transient receptor potential melastatin 2 (TRPM2) channel. Stimulating with either N-formylmethionine-leucyl-phenylalanine (fMLF) or complement protein C5a causes distinct changes of the [Na^+^]_i_. fMLF induces a sustained increase of [Na^+^]_i_, surprisingly, reaching higher values in TRPM2^−/−^ neutrophils. This outcome is unexpected and remains unexplained. In both genotypes, C5a elicits only a transient rise of the [Na^+^]_i_. The difference in [Na^+^]_i_ measured at *t* = 10 min after stimulation is inversely related to neutrophil chemotaxis. Neutrophil chemotaxis is more efficient in C5a than in an fMLF gradient. Moreover, lowering the extracellular Na^+^ concentration from 140 to 72 mM improves chemotaxis of WT but not of TRPM2^−/−^ neutrophils. Increasing the [Na^+^]_i_ by inhibiting the Na^+^/K^+^-ATPase results in disrupted chemotaxis. This is most likely due to the impact of the altered Na^+^ homeostasis and presumably NCX1 function whose expression was shown by means of qPCR and which critically relies on proper extra- to intracellular Na^+^ concentration gradients. Increasing the [Na^+^]_i_ by a few mmol/l may suffice to switch its transport mode from forward (Ca^2+^-efflux) to reverse (Ca^2+^-influx) mode. The role of NCX1 in neutrophil chemotaxis is corroborated by its blocker, which also causes a complete inhibition of chemotaxis.

## Introduction

Neutrophil migration, adhesion, neutrophil extracellular trap formation, bacterial killing, and production of reactive oxygen species (ROS) require coordinated ion fluxes (1–6). Ca^2+^ is among the best studied ions in this context (7, 8). It is a potent, multifunctional second messenger and its increase in the cytoplasm is a hallmark of neutrophil activation. An increase of the intracellular Ca^2+^ concentration ([Ca^2+^]_i_) is caused by release of Ca^2+^ from intracellular stores and by receptor- or store-operated Ca^2+^ entry (ROCE or SOCE, respectively). This relies to a large extent on transient receptor potential (TRP) and Orai channels present in the plasma membrane of neutrophils (9–13). Ca^2+^ influx was shown to be a necessary factor in neutrophil chemotaxis almost half century ago (14), but only recently the changes in [Ca^2+^]_i_ and its intracellular gradient was visualized and confirmed as a decisive factor in neutrophil chemotaxis (15). A gradient of the [Ca^2+^]_i_ was also shown to be modulated by TRPC1 channel in murine neutrophils (4). TRPC1 knock-out and its effect on Ca^2+^ homeostasis hinders neutrophil response to N-formylmethionine-leucyl-phenylalanine (fMLF).

However, an increase of the [Ca^2+^]_i_ is not the only ionic event upon cell stimulation. Neutrophil activation with fMLF leads also to an increase of the intracellular Na^+^ concentration ([Na^+^]_i_) (16). Importantly, the extracellular Na^+^ concentration ([Na^+^]_o_) also influences the [Na^+^]_i_. In normal tissue [Na^+^]_o_ equals its concentration in blood (~145 mM), but it can be lower in conditions like hypoxia (17, 18). In inflammation and tumor the [Na^+^]_o_ is usually increased (19, 20).

There are several Na^+^ transport proteins, which can account for the cell responses to changes of the extracellular ion composition or stimulation with chemoattractant. One of them is the non-selective transient receptor potential melastatin 2 (TRPM2) cation channel, which is highly expressed in neutrophils (21). Due to its presumed role as a ROS sensor, TRPM2 emerged as a putative mediator of inflammatory responses in monocytes and neutrophils (22). However, the role of TRPM2 in immune cells is rather complex (23, 24). Whether the channel has a pro- or anti-inflammatory function is still disputed, especially regarding neutrophil migratory abilities (8, 22, 23, 25). Moreover, the pH sensitivity of TRPM2 also raises the question whether the channel is still active in the acidic microenvironment of an inflammation (26–28).

Another Na^+^ transport protein, Na^+^/Ca^2+^-exchanger 1 (NCX1) stands for one of the major Ca^2+^ extrusion mechanisms in many cell types (29). It removes one Ca^2+^ in exchange for 3 Na^+^ ions transported into the cell (*forward* mode). Depending on the ion concentrations and membrane potential, it can also operate in *reverse* (Ca^2+^ influx) mode. Subtle changes of the ion composition and/or cell membrane potential are sufficient to alternate the NCX1 modes. In neutrophils, NCX1 was shown to contribute to Ca^2+^ influx (30) and membrane repolarization (31).

Finally, the activity of the NADPH-oxidase 2 (NOX2) leads to release of protons into the cytoplasm. In activated neutrophils, phosphorylation of NOX2 subunits leads to enzyme activation and assembly in the plasma membrane or in membranes of secondary granules (32, 33). Active NOX2 depolarizes the membrane through electron extrusion (34–36). The subsequent H^+^ removal is mediated not only by H^+^ channels and pumps but also by using the driving force of the Na^+^ gradient which fuels the Na^+^/H^+^- exchanger (NHE1) (37). Depolarization of the membrane also affects the electrogenic activity of the NCX1, which in effect moves one positive charge in or out of the cell affecting also Ca^2+^ flux (29). This further supports the importance of Na^+^ homeostasis in neutrophil function.

Using two *end-target* chemoattractants, fMLF (formylated peptide) and C5a (complement molecule) we analyzed neutrophil chemotaxis and changes of the [Na^+^]_i_ in neutrophils. For the first time, we show that neutrophil chemotaxis is modulated by the extra- and intracellular Na^+^ concentration. We suggest that proteins expressed in neutrophils and involved in Na^+^ homeostasis, especially NCX1, may contribute to neutrophil response upon chemoattractant stimulation. Also, knock-out of the Na^+^-permeable TRPM2 channel results in altered neutrophil [Na^+^]_i_, likely by indirectly modulating NCX1. The involvement of TRPM2 channel in neutrophil function in physiological conditions is rather minor, but may be decisive in an inflammatory environment.

## Materials and Methods

### Reagents

Reagents were purchased from SigmaAldrich®, Steinheim, Germany if not indicated otherwise.

### Animals

TRPM2^−/−^ mice were generated in a C57BL/6J background as described previously (22). TRPM2^+/+^ and TRPM2^−/−^ mice were derived from heterozygous mating. PCR using genomic DNA as template was performed to confirm the genotypes. C57BL/6J, TRPM2^+/+^ and littermate TRPM2^−/−^ mice (8–12 weeks of age) were used in the study. Mice were euthanized by cervical dislocation. Experimental protocols were approved by the local committee for animal care with permit number: 84-02.05.50.15.010.

### Cell Culture

Myelomonocytic leukemia cells (WEHI-3B) were cultured in bicarbonate-buffered Dulbecco's Modified Eagle Medium (DMEM, Merck Chemicals GmbH, Darmstadt, Germany) medium supplemented with 4 mM L-glutamine, 10% fetal bovine serum (FBS) (Gibco/ThermoFisher Scientific, Darmstadt, Germany), 100 U/ml penicillin and 100 μg/ml streptomycin (Biochrom, Berlin, Germany) at 37°C in a humidified atmosphere containing 5% CO_2_. Four days after cells reached confluence, the supernatant was collected, filtered and stored at −20°C. Since bone-marrow derived cells comprise also undifferentiated progenitors, medium from cultivated WEHI-3B cells served as a source rich in G-CSF and Il-3 and was used for neutrophil differentiation (38).

### Isolation of Murine Neutrophils

Mouse bone marrow was prepared from femurs and tibiae. Hind limbs were sterilized with 70% ethanol and dissected bones were flushed via a 0.4 mm needle with Ca^2+^/Mg^2+^-free HBSS (HBSS^−/−^, Merck Chemicals GmbH, Darmstadt, Germany) containing 25 mM HEPES and 10% FBS. Bone marrow cell suspension was dispersed and filtered with a 70 μm cell strainer and centrifuged at 4°C, 200 g for 10 min. Pelleted cells were resuspended in 1 ml HBSS^−/−^ with 25 mM HEPES and put on Histopaque®1077/1119 layers. Cells were then centrifuged at 400 *g*, for 30 min. The layer of granulocytes was taken and washed twice in HBSS^−/−^ with 25 mM HEPES and 10% FBS (centrifuged at 4°C, 200 g, 10 min). We use HBSS^−/−^ for isolating and processing the neutrophils to avoid neutrophil attaching to the labware. Cells were resuspended in bicarbonate-buffered RPMI-1640 medium with L-glutamine, 10% FBS, 10% WEHI-3B-conditioned medium, 100 U/ml penicillin and 100 μg/ml streptomycin. Cell suspension was put in 10 cm Petri dishes and incubated overnight at 37°C in a humidified atmosphere containing 5% CO_2_.

### Measurements of the [Na^+^]_i_

Ibidi μ-Slides I (ibidi GmbH, Gräfelfing, Germany) were used for sodium measurements. Slides were coated with 1 μg/cm^2^ fibronectin for 1 h at RT and washed twice with HEPES-buffered Ringer's solution (140 mM NaCl, 5.4 mM KCl, 1.2 mM CaCl_2_, 0.8 mM MgCl_2_, 10 mM HEPES, 5.5 mM glucose, pH 7.4) before the experiments. When experiments were done with a Ringer's solution with 72 or 5 mM Na^+^, we isosmotically replaced NaCl by 68 and 135 mM NMDG-Cl, respectively. Neutrophils were resuspended in 2 ml Ringer's solution and about 1 × 10^6^ cells were used for a single experiment. Cells were incubated with 5 μM Asante NaTRIUM Green-2 AM dye (ANG-2 AM) (TEFLabs Inc., Austin, USA) in Ringer's solution for 1 h at RT, put onto μ-Slides and allowed to adhere at 37°C for 10 min. The slide was washed twice with 1 ml pre-warmed Ringer's solution and mounted on the Axiovert 200 microscope (Carl Zeiss AG, Oberkochen, Germany) connected to the perfusion system, monochromator (Visitron System, Puchheim, Germany) and CCD camera (pco.edge 5.5) (PCO AG, Kelheim, Germany). A 100× oil objective (Plan Apo) was used for the observation. The filter set consisted of a T525lpxr beam splitter and an ET560/50m emission filter (Chroma Technology GmbH, Olching, Germany). Image acquisition was controlled by VisiView software (Visitron Systems GmbH, Puchheim, Germany). Excitation wavelength was set to 520 nm with 200 ms exposure time, binning 2. Images were acquired in 20 s intervals.

Experiments started after a short superfusion period with Ringer's solution until cells showed a stable fluorescent signal before the experimental solutions were applied. Where indicated, neutrophils were stimulated with 1 μM fMLF or 60 nM C5a in the superfusion solution or treated with 100 μM ouabain to inhibit the Na^+^/K^+^-ATPase and/or 10 μM KB-R7943 to inhibit the Na^+^/Ca^2+^ exchanger. Each assay lasted 10 min and was followed by a calibration. To this end, cells were sequentially superfused with solutions of different Na^+^ concentrations. Standard solutions were prepared by mixing equimolar NMDG-Cl (Na^+^-free) and Na^+^-containing solutions. 0, 10, and 20 mM Na^+^ solutions were used and ionic concentrations were verified with ABL800 blood analyzer (Radiometer Medical ApS, Brønshøj, Denmark). All calibration solutions contained 50 μM amphotericin B to permeabilize the membranes and 100 μM ouabain to inhibit the Na^+^/K^+^-ATPase. Experiments were conducted at 37°C. Acquired time-lapse stacks were analyzed using ImageJ software. After background subtraction, the total projected area and fluorescence of the cells in the field of view were analyzed (≥5 cells for each *N*; *N* = 3). The product of area and intensity was then compared with linear standard curves derived from the calibration values for each cell.

### Measurements of the [Ca^2+^]_i_

Ibidi μ-Slides I (ibidi GmbH, Gräfelfing, Germany) were prepared as described above [see section Measurements of the [Na^+^]_i_]. About 1 × 10^6^ neutrophils were used for each experiment. Cells were incubated with 3 μM Fura-2 AM (Biomol GmbH, Hamburg, Germany) for 25 min at 4°C in the dark. After incubation, neutrophils were seeded onto Ibidi μ-Slides I and incubated for 10 min in a warming cabinet and then washed with pre-warmed Ringer's solution. Measurements were performed with the same superfusion system as described above [see section Measurements of the [Na^+^]_i_]. 40× oil objective, beam splitter 400dclp and D510/40m emission filter (Chroma Technology GmbH, Olching, Germany) were used. Experiments were conducted at 37°C. Two excitation wavelengths were applied: 340 and 380 nm with 100 ms exposure time each, binning 2. Emission was measured at 510 nm. Acquisition was set for every other 20 s. Neutrophils were firstly superfused with Ringer's solution followed by Na^+^-free solution (140 mM NMDG-Cl). In next experimental phase, cells were either superfused with 1 μM fMLF only, or 1 μM fMLF with 10 μM KB-R7943. Each experiment was calibrated by applying Ringer's solution containing 1 μM ionomycin with 5 mM Ca^2+^ or Ca^2+^-free solution with 5 mM EGTA. Fluorescence intensity for each excitation wavelength for cells in the field of view was analyzed using VisiView software (Visitron Systems GmbH, Puchheim, Germany). Intensity values were background corrected and absolute values were calculated as described before (39).

### Chemotaxis Assays in a 3D Collagen Matrix

For chemotaxis assays, ibidi μ-Slides Chemotaxis 2D/3D (ibidi GmbH, Gräfelfing, Germany) were used as described previously (40). Slides were coated with 1 μg/cm^2^ fibronectin (Roche Diagnostics GmbH, Mannheim, Germany) for 1 h. Following overnight incubation cells were resuspended in HEPES-buffered Ringer's solution (140 mM NaCl or 72 mM Na^+^ and 68 mM NMDG-Cl, 5.4 mM KCl, 1.2 mM CaCl_2_, 0.8 mM MgCl_2_, 10 mM HEPES, 5.5 mM glucose; pH 7.4) and seeded in a three-dimensional collagen I (2.1 mg/ml) (Corning®, Kaiserslautern, Germany) matrix in HBSS^+/+^. The chemotactic gradients were built by adding the attractants to one of the chambers. Two *end-target* chemoattractants were used in our chemotaxis assays: fMLF (1 μM) and (recombinant murine) C5a (60 nM) (Bio-Techne GmbH, Wiesbaden-Nordenstadt, Germany). Optimal chemoattractant concentrations were chosen based on neutrophil chemotaxis indices (CI) obtained in experiments with increasing concentrations of fMLF (10, 100 nM, 1, 33 μM) and C5a (6, 60, 370 nM). Live-cell imaging was performed using phase-contrast microscopy and video cameras (models XC-ST70CE and XC-77CE) at 37°C. Using HiPic Image acquisition software (Hamamatsu Photonics, Herrsching am Ammersee, Germany) time-lapse settings were set for every 5 s for 30 min. Acquired stacks were analyzed with Amira software (Thermo Fisher Scientific, Darmstadt, Germany). Amira files were evaluated using ImageJ (National Institutes of Health, Maryland, USA) and a self-made plugin (courtesy of Peter Dieterich, Dresden). Cell velocity was calculated by applying a three-point difference quotient and CI was calculated as the migration in the direction of the chemical gradient divided by the total path length (4, 8, 40, 41).

### Reverse Transcription and Quantitative PCR (RT-qPCR)

Total RNA was isolated using TRIzol reagent and chloroform phase separation (ThermoFisher Scientific, Darmstadt, Germany). The RNA concentration was measured with a spectrophotometer and 2 μg of total RNA were reverse transcribed. SuperScript™ III Reverse Transcriptase (ThermoFisher Scientific, Darmstadt, Germany) was used for the reaction. cDNA samples were diluted in water to the final volume of 50 μl. For qPCR analysis, 2 μl of cDNA template was used in 10 μl of final reaction solution. PowerUp™ SYBR™ Green Master Mix (Applied Biosystems®/ThermoFisher Scientific, Darmstadt, Germany) was used for qPCR. We applied the following reaction protocol: an initial hold step (95°C, 10 min) and 40 cycles of a three-step amplification (95°C, 30 s; 55°C, 25 s; 72°C, 60 s). An additional melting curve stage was implemented for product control. Murine 18S rRNA transcript (m18S rRNA) served as endogenous reference. We used the following forward and reverse primers: 5′-GTA ACC CGT TGA ACC CCA TT-3′ and 5′- CCA TCC AAT CGG TAG TAG CG- 3′ (42). NCX1 primers were designed to span the region of alternative splicing specific for BD exons specific for NCX1.3 and NCX1.7 (43) (also: GenBank: AF108396.1). Forward and reverse primer sequences were: 5′- TCT CCC TTG TGC TTG AGG AAC-3′ and 5′- AGC CAC CTT TCA ATC CTC TTC T-3′. NCX1 mRNA expression was calculated from mean Ct values of technical duplicates and subtraction of mean Ct for 18S rRNA transcript (ΔCt). Values transformed to 2^−ΔCt^ were used for statistical analysis. Relative normalized transcript expression in TRPM2^−/−^ neutrophils was calculated in relation to TRPM2^+/+^ (2^−ΔΔCt^) (44).

### Statistical Analysis

All data is shown as mean ± SEM. For comparison between two groups, statistical analysis was performed using a two-tailed Student's *t*-test or Mann-Whitney *U*-test when data was not normally distributed. In experiments comparing more than 2 groups 1-way ANOVA or repeated measures ANOVA was used, with Tukey or Dunn *post-hoc* tests. For all tests, *p* < 0.05 were considered statistically significant. “*N*” stands for number of animals and “*n*” designates number of single cells analyzed.

## Results

### fMLF Stimulation Causes a Sustained Increase of the [Na^+^]_i_ in TRPM2^+/+^ and TRPM2^–/–^ Neutrophils

Like most chemoattractant receptors, fMLF receptor (FPR1) activation leads to Ca^2+^ store release and an increase of the [Ca^2+^]_i_ (45). Further Ca^2+^ influx is mediated by opening of plasma membrane channels, among others non-selective TRP channels, allowing for concurrent Na^+^ influx. TRPM2 not only allows Ca^2+^ and Na^+^ influx, but its gating mechanism requires Ca^2+^ ions and an increase of cytoplasmic ADP-ribose (46, 47). It seems that an increased [Ca^2+^]_i_ would serve as a constant positive feedback. However, at the same time, the resulting membrane depolarization dampens cation influx. The high [Ca^2+^]_i_ may also promote NCX1 forward mode in order to resolve the neutrophil activation. The activity and role of NCX1 in neutrophils, however, has not been determined unequivocally (30, 31). TRPM2 and NCX1 are highly dependent on the intra- and extracellular ionic composition. It is known that the rise of the [Ca^2+^]_i_ upon fMLF stimulation is somewhat diminished in TRPM2^−/−^ neutrophils (22, 48) which in turn may influence the NCX1 activity.

**Figure 1:**
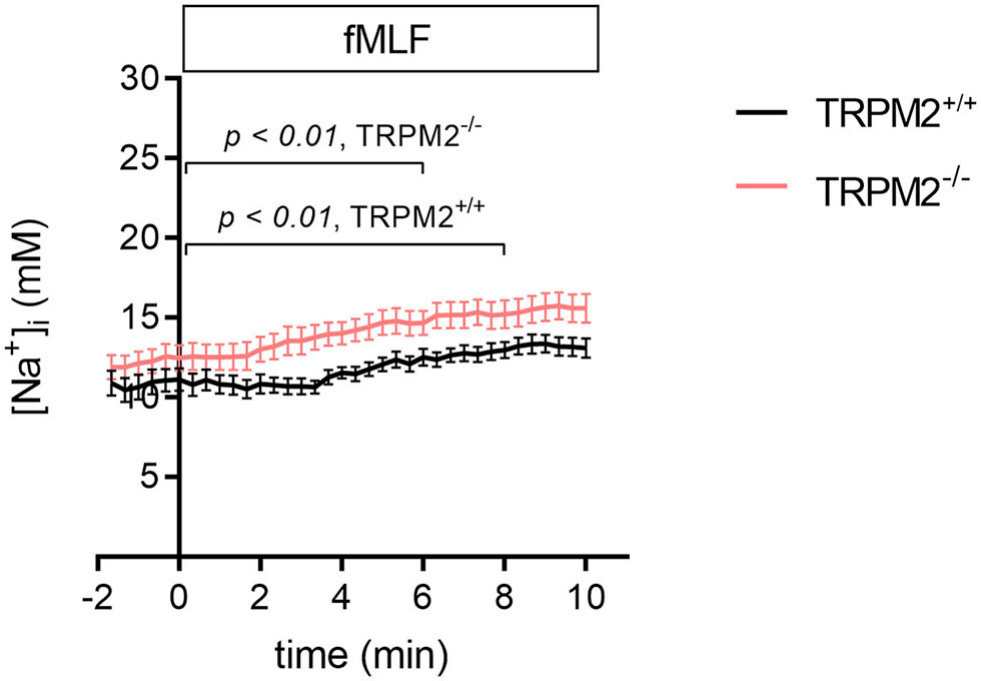
[Na^+^]_i_ upon fMLF stimulation in TRPM2^+/+^ and TRPM2^−/−^ neutrophils. fMLF stimulation leads to an increase of the [Na^+^]_i_ in neutrophils. The increase of the [Na^+^]_i_ is attenuated in the presence of TRPM2 channels. Repeated measures ANOVA was used to analyze whether time points of significant increase of the [Na^+^]_i_ differ between genotypes. It is evident that the earlier concentration rise leads to higher [Na^+^]_i_ after 10 min in TRPM2^−/−^ neutrophils. One micromolar fMLF was added at *t* = 0. For TRPM2^+/+^: *N* = 3, *n* = 23; for TRPM2^−/−^: *N* = 3, *n* = 24.

Previous experiments studying the [Na^+^]_i_ upon fMLF stimulation in human neutrophils showed an increase by about 8.5 ± 3.2 mM Na^+^ within 5 min (16). Using the novel Na^+^ indicator, ANG-2 AM, we compared the time course of changes of the [Na^+^]_i_ in murine TRPM2^+/+^ and TRPM2^−/−^ neutrophils over a 10 min time span. Knowing that TRPM2 is permeable for both Ca^2+^ and Na^+^ ions, we assumed that in the absence of the channel the increase of [Na^+^]_i_ will be hampered. However, this is not the case. A steady increase of the [Na^+^]_i_ is observed in both genotypes with the [Na^+^]_i_ reaching higher values in TRPM2^−/−^ neutrophils within 10 min (15.6 ± 0.9 vs. 13.1 ± 0.6) (Figure 1). This difference is mainly due to the earlier rise of the [Na^+^]_i_ as determined by repeated measures ANOVA. There is a significant increase of the [Na^+^]_i_ in TRPM2^−/−^ neutrophils after 6 min and only after 8 min in the presence of channel. This suggests that the channel prevents the rapid increase of the [Na^+^]_i_ during the first minute of fMLF stimulation. In the absence of the TRPM2 due to possible diminished membrane depolarization, Na^+^ influx can be facilitated and mediated through other transport proteins.

### C5a Stimulation Causes a Rapid, but Transient Increase of the [Na^+^]_i_ in TRPM2^+/+^ and TRPM2^–/–^ Neutrophils

C5a is an end-target chemoattractant and its mechanism of action after binding to C5aR resembles to a large extent the molecular pathway of FPR1 activation (45). C5aR1 also activates a phospholipase C β signaling pathway causing an increase of the [Ca^2+^]_i_ (45, 49). To our knowledge, there is no data showing the [Na^+^]_i_ in neutrophils upon C5a stimulation.

**Figure 2:**
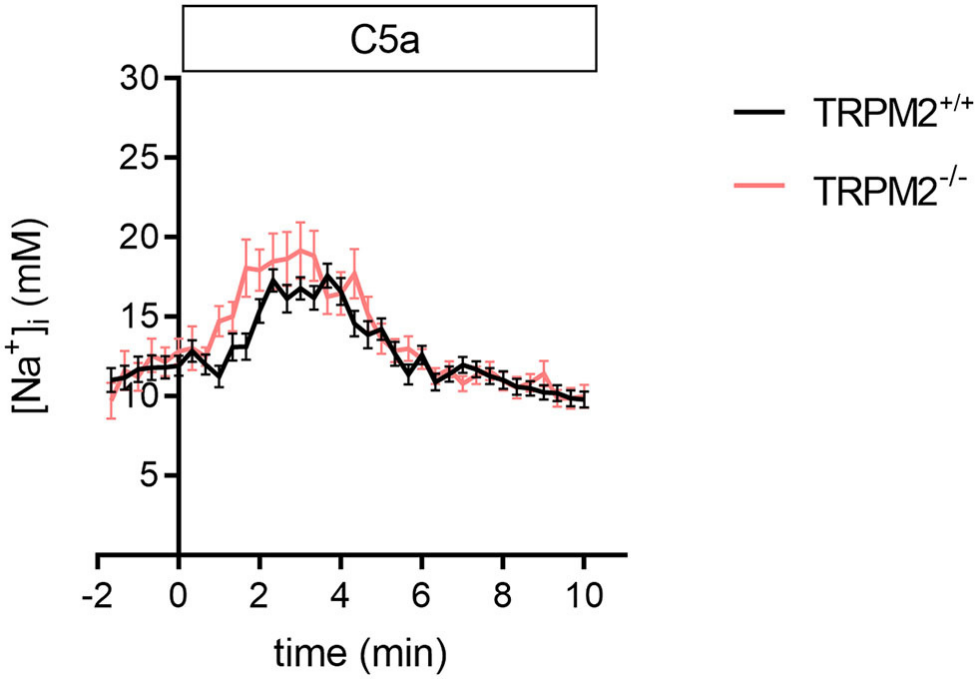
[Na^+^]_i_ upon C5a stimulation in TRPM2^+/+^ and TRPM2^−/−^ neutrophils. C5a stimulation causes a rapid and pronounced increase of the [Na^+^]_i_, but in contrast to fMLF, the [Na^+^]_i_ returns to a basal levels within 5 min. The time course of the [Na^+^]_i_ changes is similar in neutrophils of both genotypes. Sixty nanomolar C5a was added at *t* = 0. For TRPM2^+/+^ *N* = 3, *n* = 33; for TRPM2^−/−^ *N* = 3, *n* = 30.

Although C5a and fMLF employ similar signaling pathways, the temporal changes of the [Na^+^]_i_ upon C5a stimulation differ from those induced by fMLF (Figure 2). The most striking difference is that the [Na^+^]_i_ increases only transiently upon C5a stimulation. C5a causes a rapid increase of the [Na^+^]_i_ (+5.6 mM Na^+^ after 3 min) but in contrast to fMLF, also a fast recovery to the basal level (~11 mM Na^+^). The curves are similar for TRPM2^+/+^ and TRPM2^−/−^ neutrophils. There is no significant difference between the genotypes in curve slopes (only slightly faster increase in TRPM2^−/−^ neutrophils) and [Na^+^]_i_ at the end of the observation. The [Na^+^]_i_ measurements for fMLF and C5a indicate the different Na^+^ regulation upon stimulation with these chemoattractants. We therefore tested whether the differences of the [Na^+^]_i_ may directly or indirectly affect neutrophil function or reflect the fact that FPR1 and C5aR1 cause distinct ionic responses.

### Impact of Na^+^ Transport Proteins on the [Na^+^]_i_ of Neutrophils

The Na^+^/K^+^-ATPase and NCX1 are two neutrophil membrane proteins involved in Na^+^ homeostasis. The Na^+^/K^+^-ATPase sustains the Na^+^ gradient by extruding intracellular Na^+^ in exchange for extracellular K^+^ with a stoichiometry of 3:2. NCX1 is also a ubiquitous protein and has varied functions depending on cell type. Its role in activated neutrophils is not yet well-established.

Both transport proteins differ in their affinity and capacity. Constitutively active, high-affinity and low capacity Na^+^/K^+^-ATPase is less susceptible to environmental disturbances, but low-affinity high-capacity NCX1 may be critical for the cell under stress conditions. Ouabain is a widely used inhibitor of the Na^+^/K^+^-ATPase with anti-inflammatory properties. In neutrophils, ouabain diminishes CD18 expression, but the exact mechanism is not yet described (50).

KB-R7943 is a pharmacological inhibitor of the cardiac NCX1.1 splice variant in *forward* mode. However, it inhibits both *forward* and *reverse* modes of NCX1.3 and NCX1.7 splice variants, which are expressed in many non-excitable cells (IC_50_ = 2.9 and 2.4 μM, respectively) (51).

**Figure 3:**
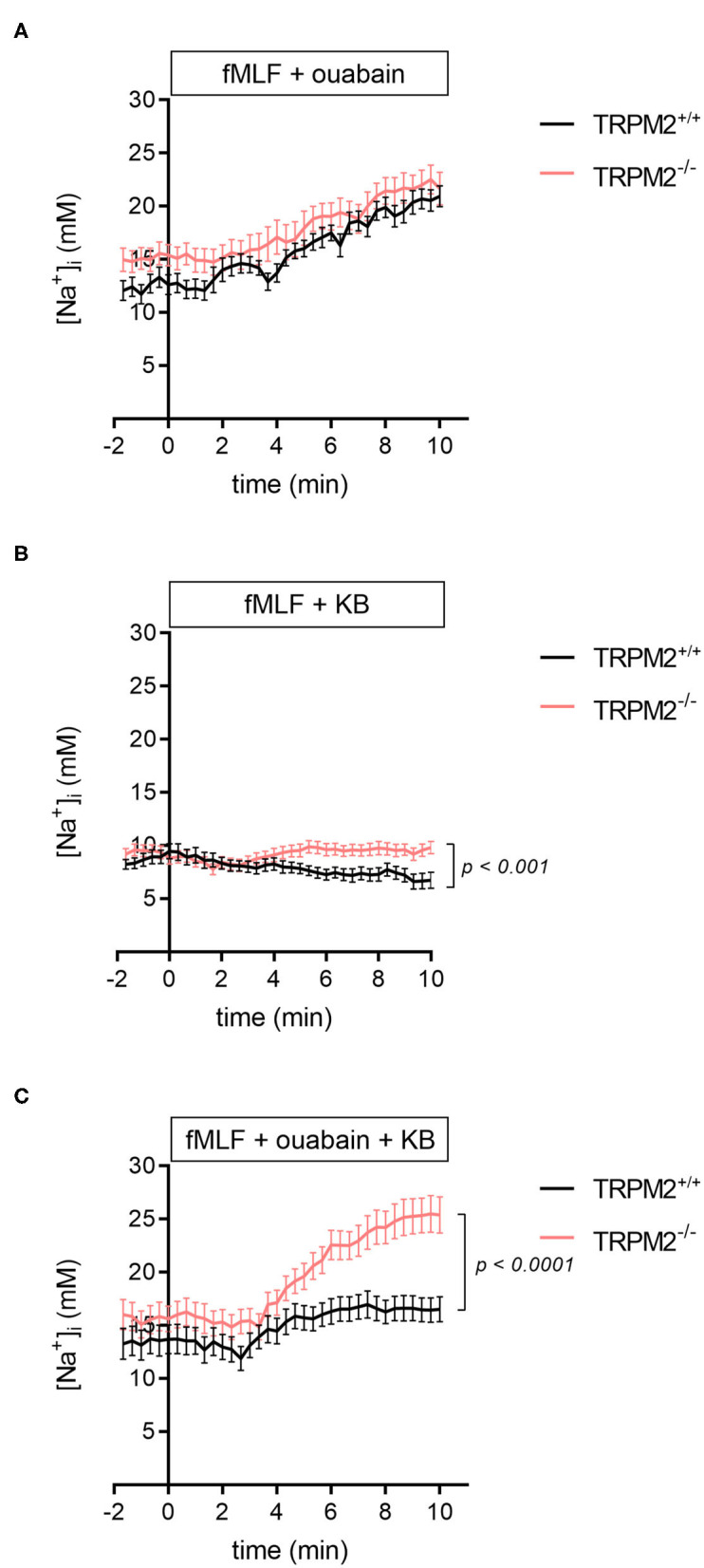
[Na^+^]_i_ upon fMLF stimulation and addition of Na^+^/K^+^-ATPase and/or NCX1 inhibitor. **(A)** In TRPM2^+/+^ and TRPM2^−/−^ neutrophils, inhibition of the Na^+^/K^+^-ATPase leads to an increase of the [Na^+^]_i_ to similar end values. **(B)** Upon the stimulation with fMLF and inhibition of NCX1, the [Na^+^]_i_ decreases in TRPM2^+/+^ but not in TRPM2^−/−^ neutrophils, reaching significantly lower values after 10 min (6.7 ± 0.8 mM vs. 9.8 ± 0.6 mM; *p* < 0.001). **(C)** Comparison of the [Na^+^]_i_ upon fMLF stimulation with Na^+^/K^+^-ATPase and NCX1 inhibitors in TRPM2^+/+^ and TRPM2^−/−^ neutrophils. Following 10 min of stimulation with fMLF, the [Na^+^]_i_ in TRPM2^+/+^ and TRPM2^−/−^ neutrophils reaches 16.5 ± 1.2 mM and 25.4 ± 1.7 mM, respectively. fMLF and inhibitors were added at *t* = 0. *N* = 3, *n* ≥ 22.

To further elucidate mechanisms underlying the [Na^+^]_i_ homeostasis in TRPM2^−/−^ neutrophils, we inhibited Na^+^/K^+^-ATPase with 100 μM ouabain and measured the [Na^+^]_i_. As expected, the [Na^+^]_i_ of fMLF-stimulated neutrophils increases to much higher values in the presence of ouabain than in the presence of fMLF alone (Figure 3A vs. Figure 1). However, there is almost no difference between TRPM2^+/+^ and TRPM2^−/−^ neutrophils (20.9 ± 1 mM vs. 21.7 ± 1.5 mM). This increase confirms the activity of the Na^+^/K^+^-ATPase pump, which is responsible for extruding Na^+^ from the cytosol of neutrophils.

Using KB-R7943 alone abolishes the fMLF-induced rise of the [Na^+^]_i_. In TRPM2^+/+^ neutrophils, the NCX1 inhibitor lowers the final sodium concentration (Figure 3B). In the absence of TRPM2 channels, fMLF stimulation and NCX1 inhibition lead to a higher final [Na^+^]_i_. The time course of the [Na^+^]_i_ changes is consistent with NCX1 operating in the forward mode in TRPM2^+/+^ neutrophils during the entire observation period. Initially, TRPM2^−/−^ neutrophils behave identically. However, there is no further decrease of the [Na^+^]_i_ in TRPM2^+/+^ neutrophils. This temporal distinction in neutrophil [Na^+^]_i_ may suggest altered NCX1 function in neutrophils lacking TRPM2 channel.

When neutrophils are superfused with a combination of 1 μM fMLF, 100 μM ouabain and the NCX1 inhibitor KB-R7943 (10 μM), the [Na^+^]_i_ in TRPM2^+/+^ neutrophils is lower than with fMLF and ouabain only (from 20.9 ± 1 mM down to 16.5 ± 1.2 mM). In contrast, in TRPM2^−/−^ neutrophils the [Na^+^]_i_ rises even higher (from 21.7 ± 1.5 mM up to 25.4 ± 1.7 mM) (Figure 3C). This further supports different activity of NCX1 in TRPM2^+/+^ and TRPM2^−/−^ neutrophils, however, may also indicate the compensatory action of other Na^+^ transport protein.

### Decrease of Extracellular [Na^+^] Causes a Decrease of the Neutrophil Intracellular [Na^+^]

**Figure 4:**
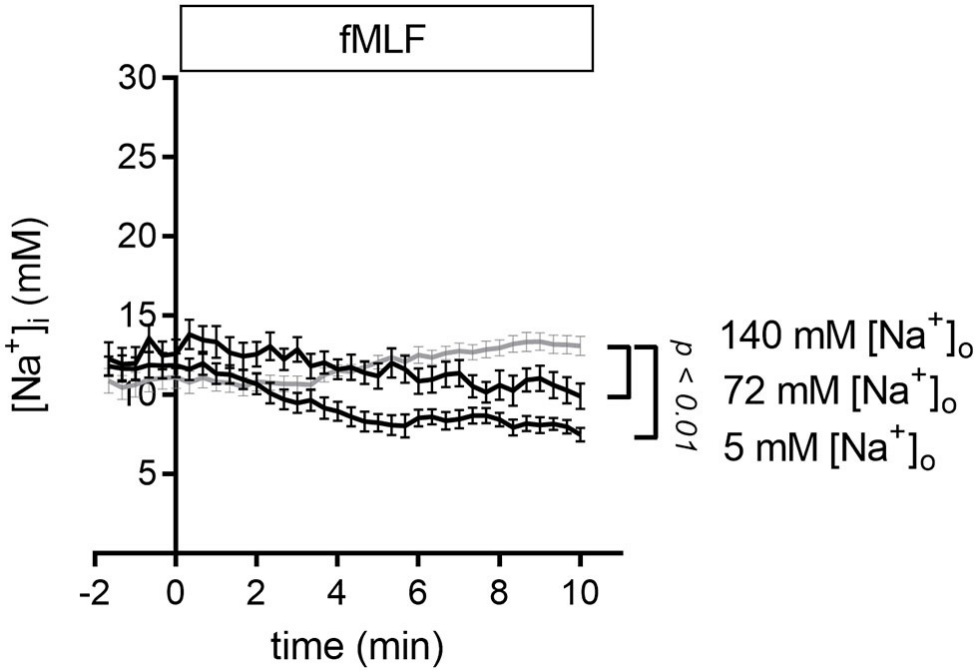
[Na^+^]_i_ upon fMLF stimulation in Ringer's solutions with reduced Na^+^ concentrations. Stimulation of neutrophils with fMLF in Ringer's solution with reduced extracellular [Na^+^] leads to a decrease of the [Na^+^]_i_. For comparison, we replotted the results of TRPM2^+/+^ neutrophils stimulated in the presence of 140 mM Na^+^ (gray curve) already depicted in Figure 1. fMLF and solutions of low Na^+^ were added at *t* = 0. *N* = 3, *n* ≥ 23.

To analyze the impact of decreasing the [Na^+^]_o_ we measured the [Na^+^]_i_ of neutrophils that were stimulated with fMLF in Ringer's solution with reduced Na^+^ concentration. Lowering the [Na^+^]_o_ to 72 or 5 mM causes a fall of the [Na^+^]_i_ compared to the control situation (Figure 4). One of the possible explanations of these results is that less Na^+^ is entering the cell via NCX1 activity so that the extrusion of the Na^+^ ions dominates.

### Removal of Extracellular Na^+^ Causes an Increase of the Neutrophil [Ca^2+^]_i_

**Figure 5:**
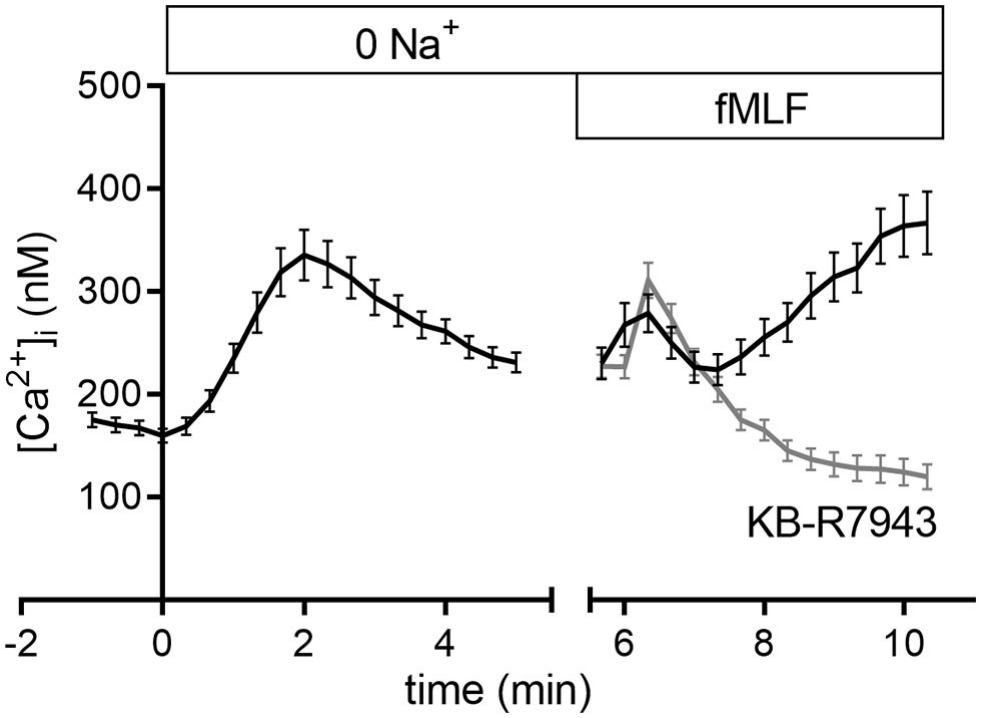
Intracellular Ca^2+^ concentration in nominally Na^+^-free Ringer's solution upon fMLF stimulation with or without NCX1 inhibition. The increase of the [Ca^2+^]_i_ upon removal of extracellular Na^+^ indicates the expression and activity of the NCX1. Subsequent stimulation in Na^+^-free solution leads to a transient increase of the [Ca^2+^]_i_ followed by sustained rise. Concurrent inhibition of the NCX1 prevents this increase of the [Ca^2+^]_i_. *N* = 3; *n* ≥ 100.

To further analyze NCX function and elucidate the impact of extracellular Na^+^ on the intracellular Ca^2+^ concentration, we superfused neutrophils with a nominally Na^+^-free Ringer's solution and measured the [Ca^2+^]_i_ (Figure 5). The nominal removal of extracellular Na^+^ leads to an increase of the [Ca^2+^]_i_ which is indicative of NCX1 activity in neutrophils. Subsequent stimulation with fMLF induces a biphasic increase of the [Ca^2+^]_i_. A short rapid peak is followed by a subsequent slow increase of the [Ca^2+^]_i_. However, inhibition of the NCX1 prevents the rise of the [Ca^2+^]_i_ and rather leads to a decrease of the [Ca^2+^]_i_. This suggests that in a nominally Na^+^-free solution NCX1 is active in the *reverse* mode.

### C5a Causes More Robust Chemotaxis of Murine neutrophils Than fMLF

One of the pivotal neutrophil functions is to rapidly reach the site of injury or inflammation. The ability to follow chemoattractant gradients can be measured *in vitro* using various migration assays. In our experimental setting, neutrophils were exposed to chemoattractant (1 μM fMLF or 60 nM C5a) gradients after they had been embedded within a 3D collagen I matrix, to mimic their physiological environment in a tissue.

**Figure 6:**
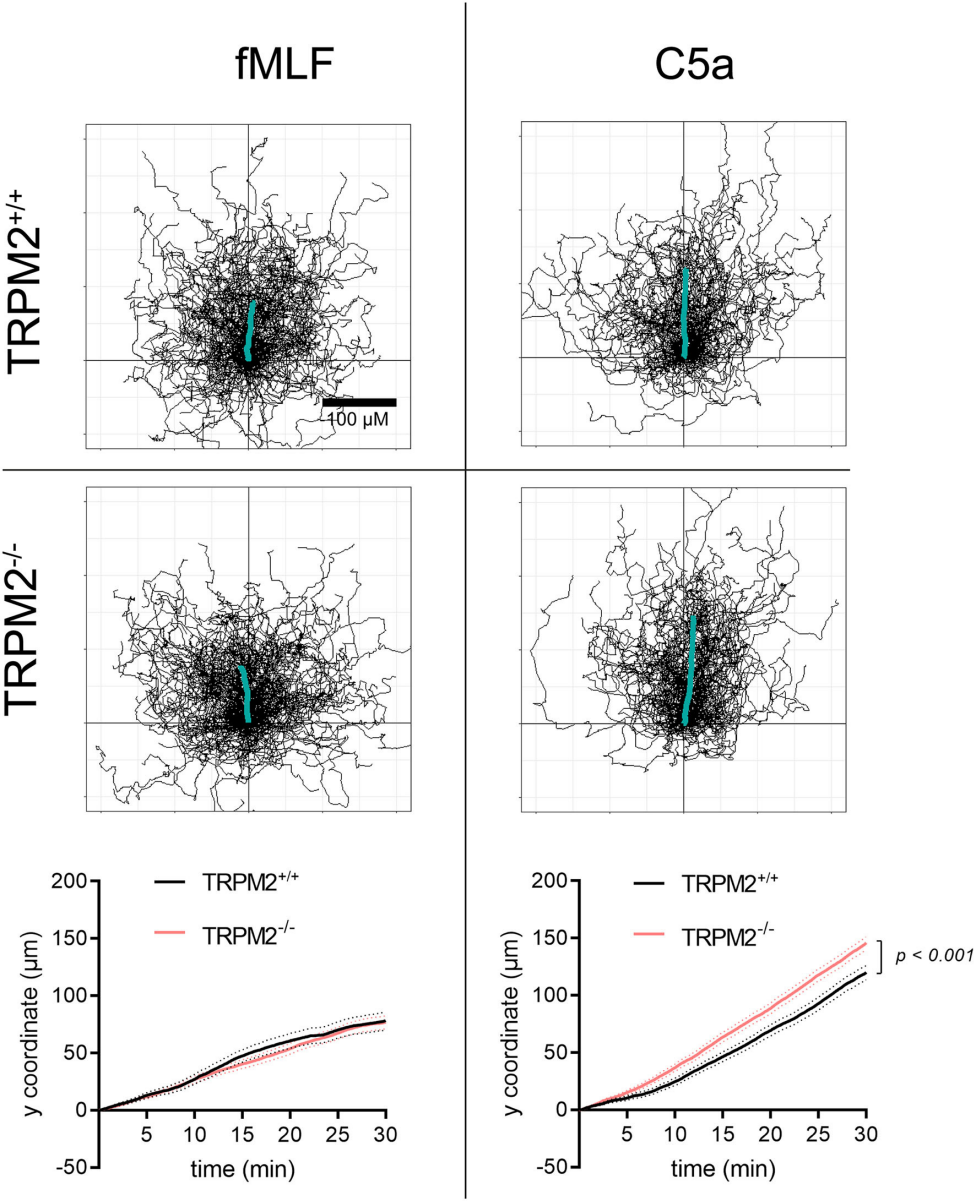
Trajectories of TRPM2^+/+^ and TRPM2^−/−^ neutrophils in fMLF and C5a gradients. fMLF induces less robust chemotaxis than C5a as shown by trajectories and mean distances covered toward the chemoattractants. Trajectories are normalized to common starting points, and the thick lines represent the averaged cell path of the entire cell populations. There are no differences between genotypes in the fMLF gradient. In a C5a gradient, TRPM2^−/−^ neutrophils have a higher chemotaxis index (not shown) and cover longer distances toward the chemoattractant (fMLF: TRPM2^+/+^, *N* = 7, *n* = 140, TRPM2^−/−^, *N* = 8, *n* = 160; C5a: TRPM2^+/+^, TRPM2^−/−^, *N* = 6, *n* = 120).

fMLF induces directional migration of neutrophils to a lesser extent than C5a (Figure 6). Both chemoattractants are used in previously established optimal concentrations (data not shown). Cell trajectories and the distance covered toward fMLF over time differ from those in a C5a gradient. Chemotaxis toward C5a is not only more prominent, but also different between the genotypes. TRPM2^−/−^ neutrophils follow the C5a gradient slightly better than TRPM2^+/+^. This difference, not revealed in our previous studies using three mice of each genotype (8), became apparent after more than doubling the number of mice analyzed. It supports the view that TRPM2 mitigates neutrophil migration (25).

### Lowering the [Na^+^]_o_ Augments Neutrophil Chemotaxis in fMLF Gradient

Assuming that the differences in neutrophil response may depend on the Na^+^ homeostasis, we analyzed neutrophil chemotaxis in an fMLF gradient in an isosmotic, low sodium (72 mM Na^+^) solution. Under these conditions, the neutrophils adapt by lowering their [Na^+^]_i_; the [Na^+^]_i_ reaches only ~10 mM as compared to 13.1 ± 0.6 mM when using the regular Ringer's solution (see Figure 4). It is known that lowering of the [Na^+^]_o_ does not affect the number of FPR1 receptors in the membrane, but may increase receptor affinity (52). To elucidate whether lower extracellular Na^+^ is beneficial for neutrophil chemotaxis we compared the chemotaxis index (CI) of fMLF-stimulated neutrophils in 140 and 72 mM [Na^+^]_o_.

**Figure 7:**
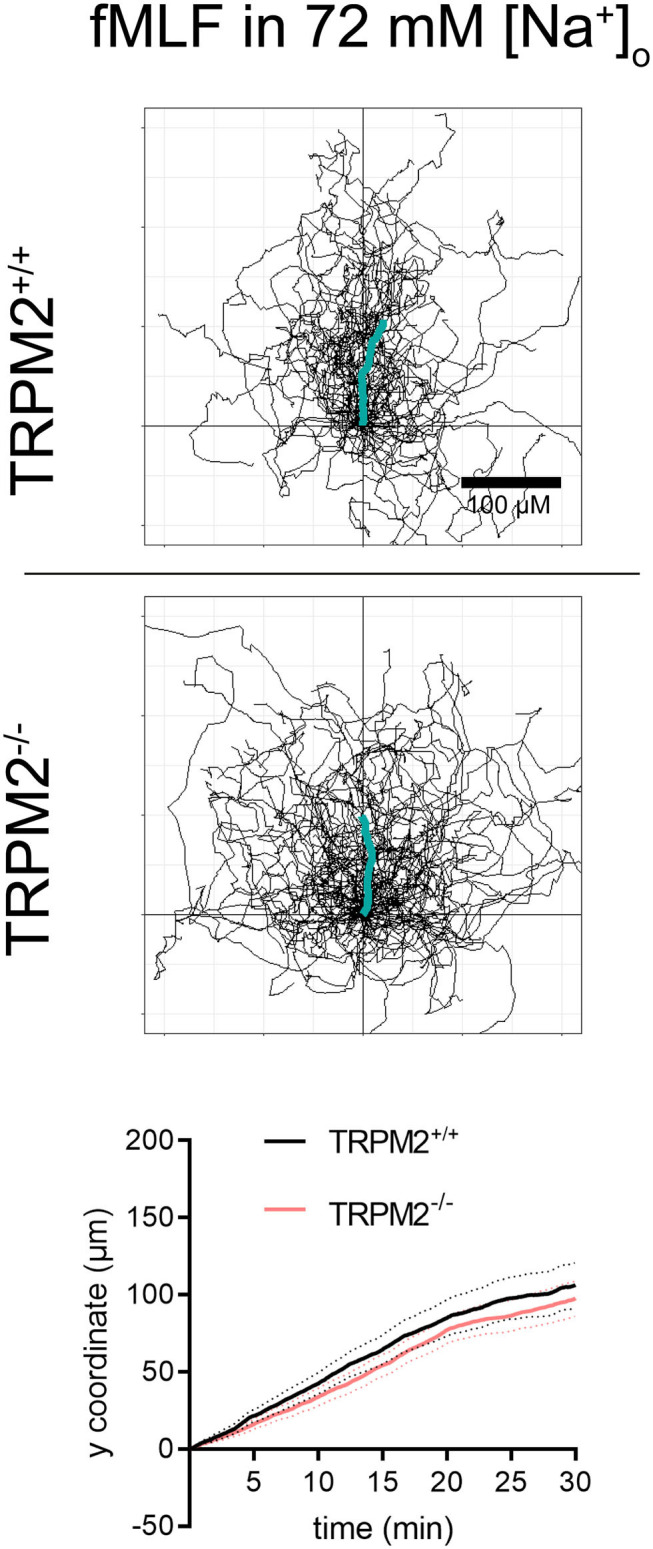
Neutrophil migratory parameters in an fMLF gradient and 72 mM extracellular sodium. Cell trajectories and mean distances covered toward fMLF (TRPM2^+/+^, *N* = 3, *n* = 60, TRPM2^−/−^, *N* = 4, *n* = 80).

**Figure 8:**
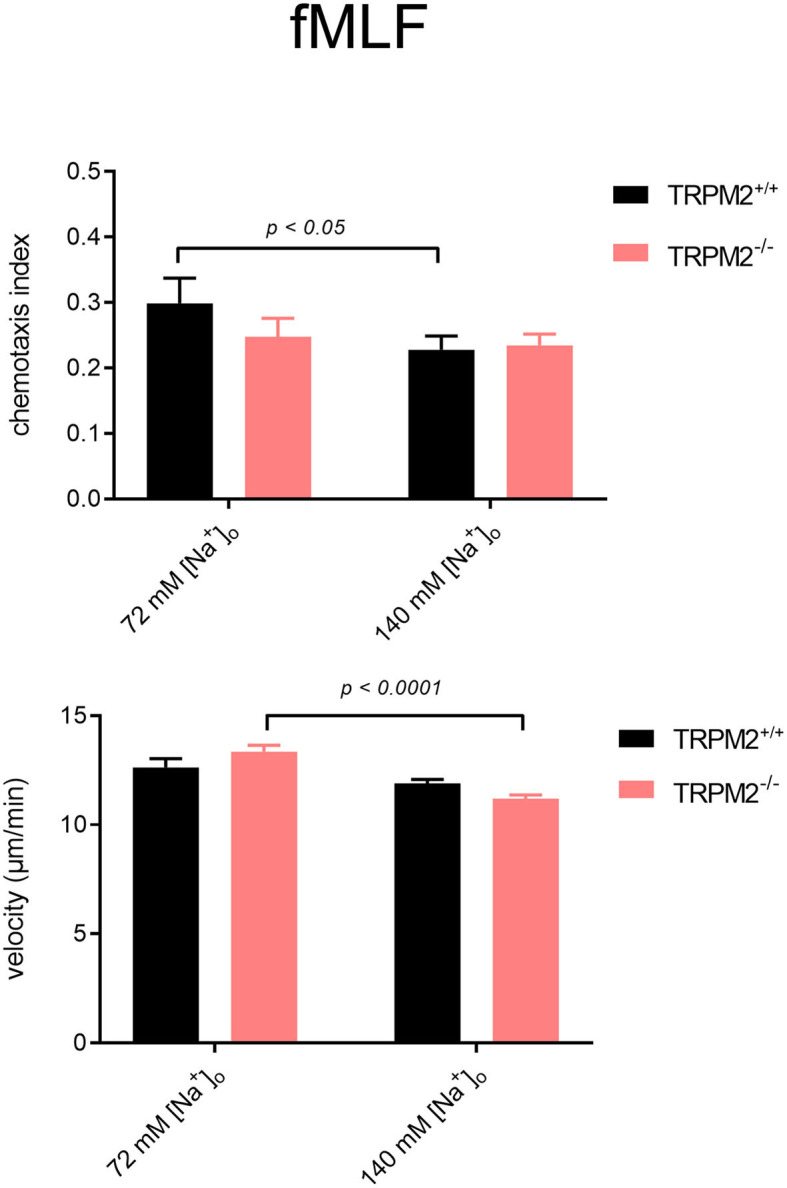
Neutrophil chemotaxis indices and velocity upon stimulation with fMLF in different [Na^+^]_o_. Comparison of chemotaxis indices and mean velocities in low and physiological [Na^+^]_o_. In the lower sodium concentration, TRPM2^+/+^ neutrophils follow the fMLF gradient better, but TRPM2^−/−^ neutrophils, despite increased velocity, show no improvement in chemotaxis (TRPM2^+/+^, *N* = 3, *n* = 60, TRPM2^−/−^, *N* = 4, *n* = 80).

TRPM2^+/+^ neutrophils follow the fMLF gradient better when the Na^+^ concentration is reduced to 72 mM (Figures 7, 8). The CI of TRPM2^+/+^ is increased in solution of reduced Na^+^, TRPM2^−/−^ neutrophils, however, cover longer distances, which is evident by more “spread” trajectories (Figure 7) and higher velocity (Figure 8). These results indicate that in the presence of the TRPM2 channel, lower [Na^+^]_i_ improves neutrophil directionality. However, in the absence of the channel, only random cell migration is increased.

### Blocking the Na^+^/K^+^-ATPase and NCX1 Restrains Neutrophil Chemotaxis

**Figure 9:**
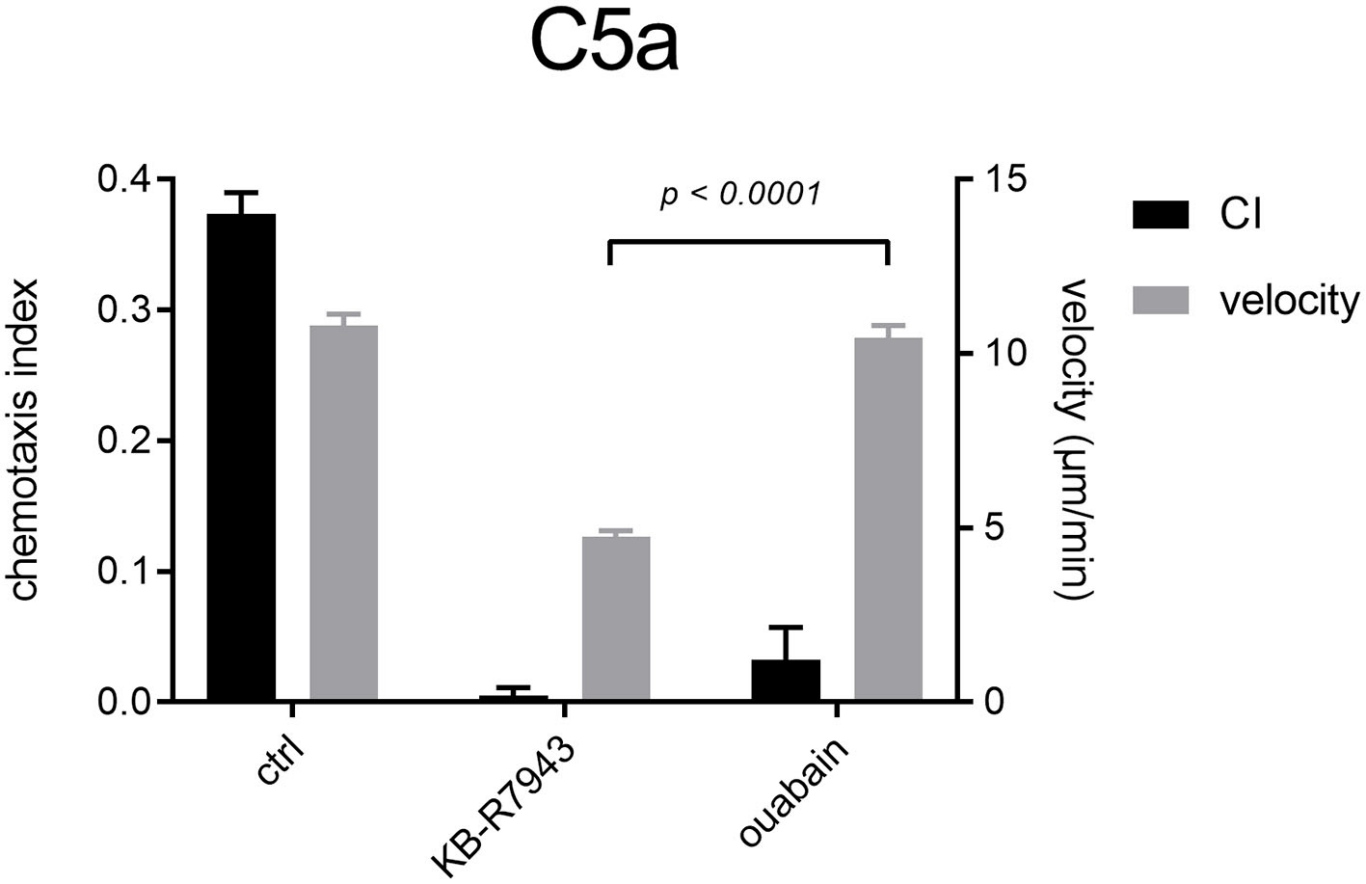
Neutrophil chemotaxis indices and velocity upon stimulation with C5a and inhibition of Na^+^/K^+^-ATPase or NCX1. Na^+^/K^+^-ATPase and NCX1 inhibitors almost completely abrogate neutrophil chemotaxis. Inhibiting NCX1 with KB-R7943 affects neutrophil velocity much stronger than ouabain. For clarity, only the significant difference between velocity values using inhibitors is shown (*N* = 3, *n* = 60).

To analyze the consequences of the increased [Na^+^]_i_ in C5a-stimulated neutrophils, we inhibited the Na^+^/K^+^-ATPase of neutrophils with 100 μM ouabain (Figure 9). Inhibition of the Na^+^/K^+^-ATPase diminishes neutrophil chemotaxis. Surprisingly, mean velocity values are very similar to those upon C5a stimulation only (ctrl). Addition of 10 μM KB-R7943 strongly impedes neutrophil chemotaxis and velocity.

### NCX1 Splice Variants Containing BD Exons Are Expressed in Neutrophils

**Figure 10:**
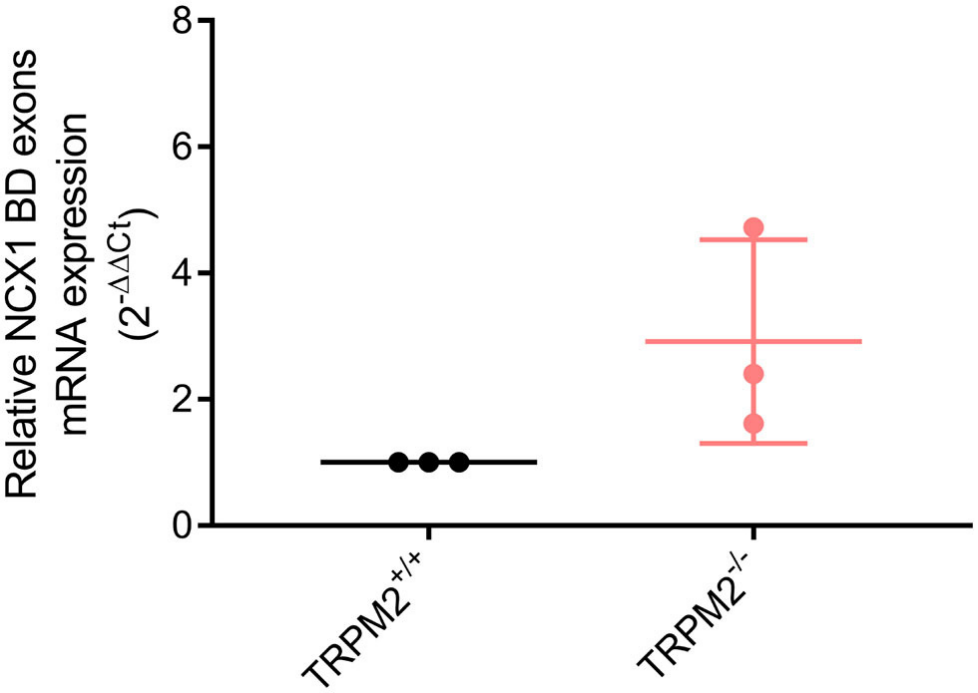
mRNA expression of NCX1 region of alternative splicing containing BD exons. NCX1 splice variants sensitive to inhibition with 10 μM KB-R7943 in reverse and forward mode are expressed in neutrophils (*N* = 3).

KB-R7943 was developed as a reverse mode NCX1 inhibitor, although it can also inhibit the forward mode. The IC_50_ of forward mode inhibition depends on the NCX1 splice variants (51, 53). Splice variants containing BD exons [predominantly NCX1.3, NCX1.7 (51)] are almost completely inhibited in the forward mode by the concentration used in our [Na^+^]_i_ measurements (10 μM KB-R7943). To confirm the presence of NCX1 splice variants sensitive to inhibition of both modes, we analyzed the expression of BD exons by means of qPCR. As shown in Figure 10, the BD transcript is expressed in both neutrophil genotypes. Expression values calculated by housekeeping gene Ct value subtraction and conversion to 2^−ΔCt^ expression level do not differ (*p* = 0.19, data not shown) and normalized relative expression (2^−ΔΔCt^) is not different in neutrophils from both genotypes.

### [Na^+^]_i_ Is a Factor Involved in Neutrophil Chemotaxis

**Figure 11:**
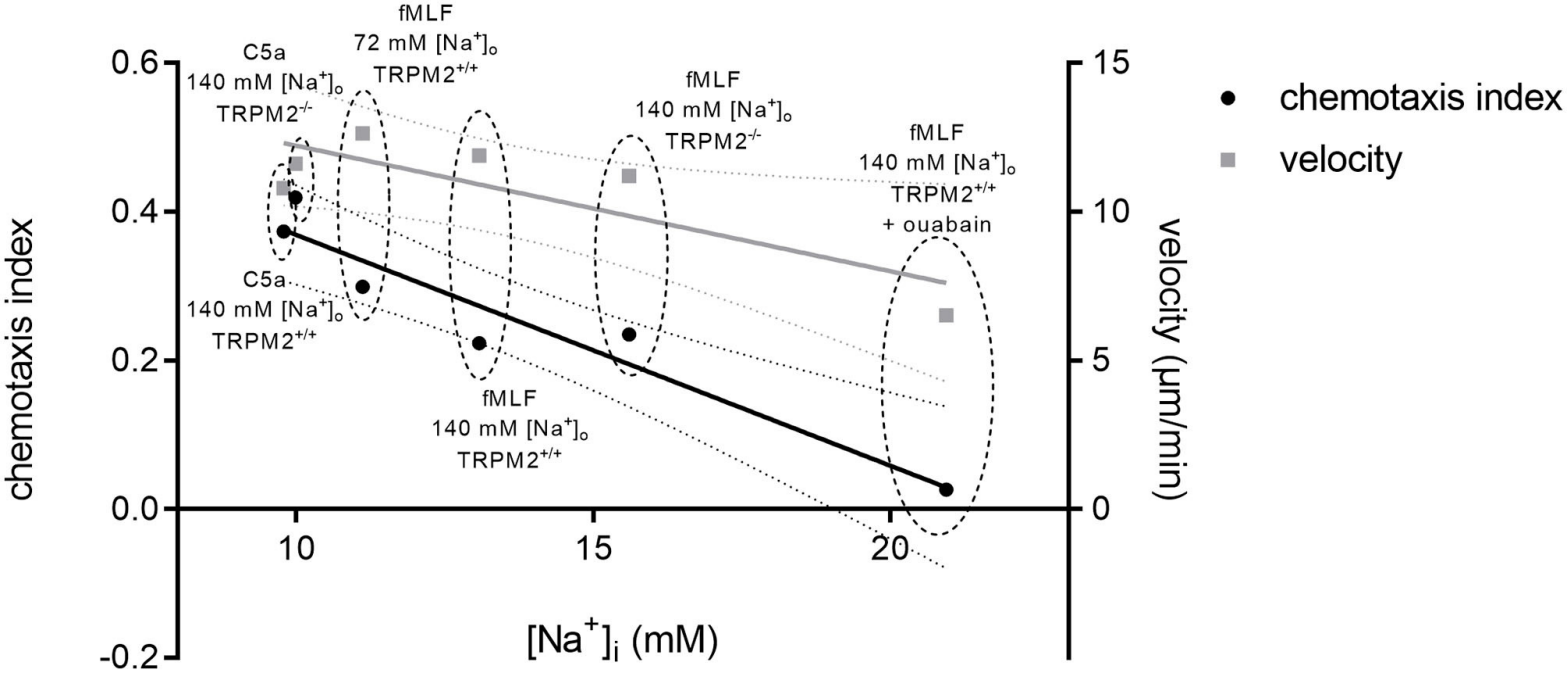
Chemotaxis and mean velocity values plotted against the respective [Na^+^]_i_ after 10 min of chemoattractant stimulation. Summary of [Na^+^]_i_ and chemotaxis index in TRPM2^+/+^ and TRPM2^−/−^ neutrophils following stimulation with fMLF or C5a. The curves reveal an inverse correlation between the [Na^+^]_i_ and neutrophil chemotaxis (Pearson's correlation coefficient (*r* = −0.9584; *p* = 0.0026)). Neutrophil velocity seems to be affected to a lesser extent by [Na^+^]_i_ (with Pearson's correlation coefficient (*r* = −0.8307; *p* = 0.0405)). Dotted lines represent confidence intervals (95%). CI and velocity values from respective assays are encircled and described.

Finally, taking under consideration all chemotaxis assays and [Na^+^]_i_ measurements performed under similar conditions, we observed a correlation between the [Na^+^]_i_ and neutrophil chemotaxis index (Figure 11). The higher [Na^+^]_i_ is associated with lower CI. In contrast, there is only a minor impact of the [Na^+^]_i_ on cell velocity. Thus, chemotaxis appears to be exquisitely sensitive to changes of the [Na^+^]_i_ which in neutrophils is controlled by Na^+^-transport proteins presumably, to the large extent by NCX1.

## Discussion

The intracellular Na^+^ homeostasis in neutrophils is tightly coupled with Ca^2+^ and H^+^ fluxes. Since the concentrations of these ions change drastically when neutrophils are activated, it is not a surprise that Na^+^-transport proteins also play a role in neutrophil function.

Studies on NCX1 in neutrophils show that the exchanger partially contributes to Ca^2+^ influx in activated neutrophils (30, 31). Membrane potential as well as the extra- and intracellular concentrations of Na^+^ and Ca^2+^ ions determine the NCX1 transport mode. Neutrophil stimulation or the harsh inflammatory extracellular environment cause alterations of the above-mentioned variables. Importantly, only slight changes of the [Na^+^]_i_ in neutrophils or of their membrane potential, determine NCX1 mode and thus, its consequence for Na^+^/Ca^2+^ homeostasis. Assuming the neutrophil resting membrane potential to be at −60 mV (54), a [Na^+^]_o_ of 140 mM and a free intra-/extracellular [Ca^2+^] of 100 nM/1.2 mM, respectively, an increase of the [Na^+^]_i_ from 12 to 13 mM results in a driving force for NCX1 *reverse* mode as calculated from ΔG_NCX_ (55). At [Na^+^]_i_ = 11 mM, a depolarization of the membrane potential to−47 mV also leads to mode switch (29). In our studies, we show that neutrophil [Na^+^]_i_ is remarkably close to the values which determine NCX1 modes. Therefore, we presume that already slight differences of the [Na^+^]_i_ in TRPM2^+/+^ and TRPM2^−/−^ neutrophils may in some circumstances affect neutrophil behavior, likely due to altered NCX-mediated Ca^2+^ influx. The contribution of NCX to Ca^2+^ influx may become even more important during the respiratory burst of primed, maximally stimulated neutrophils when their membrane potential may be as high as +60 mV (56).

Despite the fact that Na^+^ is the electrolyte with the highest concentration in the plasma and the interstitial fluid, there is only scarce data on the Na^+^ homeostasis in neutrophils. Previous studies using neutrophils and differentiated HL-60 cells have shown a rise of the [Na^+^]_i_ upon fMLF stimulation. Researchers concluded that influx of both Ca^2+^ and Na^+^ is pivotal for neutrophil exocytosis and production of ${\text{O}}_{2}^{-}$ (16, 57). In many cell types, an increased [Na^+^]_i_ is associated with pathological conditions like ischemia and excessive ROS production (58), but the consequences of the [Na^+^]_i_ rise for neutrophil chemotaxis were left unexplored.

By elucidating the changes of the [Na^+^]_i_ in neutrophils, we may now suggest that Na^+^ transport proteins contribute to alternations in [Ca^2+^]_i_ and neutrophil chemotaxis. In this context it was intriguing to observe that the [Na^+^]_i_ rises more strongly in TRPM2^−/−^ than in WT neutrophils following fMLF stimulation.

Putative explanation could be based on the TRPM2 involvement in the regulation of neutrophil membrane potential. We can hypothesize that upon fMLF stimulation, the lack of the channel renders neutrophil membrane permissive for Na^+^ influx, but it is not the case upon C5a stimulation. Chemoattractant's different impact on the neutrophil membrane potential may result in somewhat surprising outcomes.

Unfortunately, the unexpected [Na^+^]_i_ rise upon fMLF stimulation in TRPM2^−/−^ neutrophils remains unexplained. Also, there is no impact of the higher [Na^+^]_i_ on TRPM2^−/−^ chemotaxis in fMLF gradient. The prominent increase of [Na^+^]_i_ upon KB-R7943 and ouabain treatment in TRPM2^−/−^, but not in WT neutrophils suggests altered NCX1 activity, however this assumption needs to be corroborated. Upon C5a stimulation, despite similar [Na^+^]_i_ courses, TRPM2^−/−^ neutrophils migrate further in chemoattractant direction. The complexity of mechanisms allow us only to speculate and the detailed explanation requires additional studies. Our ongoing and future analysis of absolute neutrophil membrane potential values may shed some light on this matter. We presume that channel knock out results in altered membrane potential and/or compensatory activities of other ion transport proteins (presumably NCX1). Worth noting, TRPM2^−/−^ neutrophils often display slightly higher basal [Na^+^]_i_. Whether this is caused by permissive resting membrane potential or for e.g., different basal NCX1 activity is yet to be explored.

Despite the outlying results of the neutrophils lacking TRPM2 channel, in our view, Na^+^ indirectly affects neutrophil migration (*via* Ca^2+^ modulation) and may directly and specifically affect neutrophil chemotaxis (directed migration).

Two chemoattractants, fMLF and C5a, differently influence the [Na^+^]_i_ and neutrophil chemotaxis, supporting our assumptions about a role of Na^+^ ions in directed migration of neutrophils. A sustained increase of the [Na^+^]_i_ upon fMLF stimulation correlates with worse chemotaxis. On the contrary, C5a induces only a transient increase of the [Na^+^]_i_ with rapid recovery to the basal [Na^+^]_i_, which coincides with efficient chemotaxis. The notion that the [Na^+^]_i_ is more critical for chemotaxis rather than for migration itself is supported by the fact that inhibiting the Na^+^/K^+^-ATPase abolishes neutrophil chemotaxis almost completely, but has a much lesser impact on neutrophil velocity. Thus, despite obvious Na^+^ influence on Ca^2+^ homeostasis, we suggest the distinct roles of Na^+^ and Ca^2+^ ions in neutrophil function. We propose that Na^+^ gradients may strongly influence neutrophil directionality, while Ca^2+^ is pivotal for neutrophil migration.

This leads to another, yet still quite speculative explanation for the importance of the Na^+^ homeostasis for neutrophil chemotaxis. It relates to the fact that type A GPCRs including chemoattractant receptors are allosterically inhibited by Na^+^ bound to the receptor protein. GPCRs are constitutively active, but Na^+^ ions keep the receptors in an inactive state. This was also shown for FPR and C5R1 where Na^+^ ions act as an allosteric inverse agonist (59). Upon binding of the ligand, e.g., fMLF, Na^+^ has to leave its binding site in the GPCR in order to allow its full activation. One could therefore speculate that local changes of the Na^+^ concentration and thereby changes of the transmembranous Na^+^ gradient can modulate the activity of chemoattractant receptors (60). However, so far it is unknown whether the transmembranous Na^+^ gradient and/or the cell membrane potential contribute to this unbinding step. If this were the case, our results could be interpreted such that the lower [Na^+^]_i_ following the stimulation with C5a activation would help to remove allosteric inhibition and allow a stronger receptor activation than after stimulation with fMLF. Consequently, chemotaxis toward C5a is more efficient.

In our view the [Na^+^]_i_ impacts indirectly on neutrophil migration and chemotaxis by regulating the NCX1. When NCX1 is inhibited, neutrophil migration is severely impaired. A likely explanation for the importance of the NCX1 function is its role in maintaining the proper gradients of the intracellular Ca^2+^ concentration that are found in many migrating cells including neutrophils (4, 5). However, the use of inhibitors has several caveats. KB-R7943, a presumed inhibitor of NCX1, was shown to have off-targets. At concentrations used in our studies, KB-R7943 also may inhibit TRPC channels expressed in neutrophil such as TRPC3 and TRPC6. This leads to diminished Ca^2+^ influx, what can be partially the cause of the [Ca^2+^]_i_ drop upon KB-R7943 addition to fMLF-stimulated neutrophils as shown in Figure 5. In astrocytes, KB-R7943 was also shown to inhibit SOCE (61). With these assumptions, we cannot dismiss the possibility that the used inhibitor affects neutrophil migration through several targets. On the other hand, we showed previously that Ca^2+^ signaling of murine neutrophils is independent of TRPC6 channels (40) and that TRPC3 channel mRNA is expressed only at a very low level (4). Moreover, ion substitution assays as well as the measurements of the [Na^+^]_i_ and the [Ca^2+^]_i_ provide strong support for NCX1 expression and activity in neutrophils and show that Na^+^ influences [Ca^2+^]_i_ and neutrophil response. The novel assumption derived from our studies is that although Na^+^ fluxes (or the NCX1 activity) affect important Ca^2+^ homeostasis, the former seems to be pivotal for the *directed* neutrophil migration. Moreover, according to our findings, altered [Na^+^]_o_ in inflammation and tumor environment may be considered as a factor, which (even if indirectly) modulates neutrophil migration and chemotaxis.

Besides Na^+^ and Ca^2+^ transport, neutrophil activation induces H^+^ flux. Transient neutrophil intracellular acidification and depolarization are due to NOX2 activity and resolved predominantly by H_v_1 channel (62, 63). However, another Na^+^-dependent exchanger, NHE1, contributes to the electroneutral removal of H^+^. NHE1 was shown to be involved in production of arachidonic acid derivatives in neutrophils (64) and polarization of migrating cells (65, 66). Our own studies show that NHE1 contributes to neutrophil chemotaxis and regulation of intracellular pH (pH_i_) (unpublished data). This Na^+^-dependent pH_i_ regulation has to be considered when interpreting the [Ca^2+^]_i_ changes upon Na^+^ removal. The anticipated decrease pH_i_ may have caused an inhibition of NCX1 and thereby caused an only transient increase of the [Ca^2+^]_i_ shown in Figure 5.

Taken together, our study shows that TRPM2 channels have a subtle impact on neutrophil migration and chemotaxis by paradoxically regulating the [Na^+^]_i_ of neutrophils. However, the exact mechanism of [Na^+^]_i_ regulation by TRPM2, requires further studies. On the other hand, NCX1 activity and its driving force, the transmembranous Na^+^ gradient, appears to be an important factor for neutrophil function. The complexity of the mechanisms in which Na^+^ is involved, does not allow for simple assumptions. However, the present and future studies will help to understand the interwoven aspects of ionic homeostasis in activated neutrophils.

## Data Availability Statement

The datasets generated for this study are available on request to the corresponding author.

## Ethics Statement

The animal study was reviewed and approved by Landesamt für Natur, Umwelt und Verbraucherschutz NRW permit number: 84-02.05.50.15.010.

## Author Contributions

KN, MR, ML, JS, LO, SSc, SSa, ZP, and EB performed the experiments. KN made statistical analyses. KN and AS designed the study and wrote the manuscript. All authors edited the manuscript and approved the submitted version.

## Conflict of Interest

The authors declare that the research was conducted in the absence of any commercial or financial relationships that could be construed as a potential conflict of interest.
